# PAI1 is a Marker of Bad Prognosis in Rectal Cancer but Predicts a Better Response to Treatment with PIM Inhibitor AZD1208

**DOI:** 10.3390/cells9051071

**Published:** 2020-04-25

**Authors:** Sandra Muñoz-Galván, Maria Rivero, Javier Peinado-Serrano, Julia Martinez-Pérez, MC Fernández-Fernández, María José Ortiz, José M. García-Heredia, Amancio Carnero

**Affiliations:** 1 Instituto de Biomedicina de Sevilla, IBIS, Hospital Universitario Virgen del Rocío, Universidad de Sevilla, Consejo Superior de Investigaciones Científicas, Avda. Manuel Siurot s/n, 41013 Seville, Spain; smunoz-ibis@us.es (S.M.-G.); mariariverosilva@hotmail.com (M.R.); jvrr18@gmail.com (J.P.-S.); julia0802@hotmail.com (J.M.-P.); jmgheredia@us.es (J.M.G.-H.); 2 CIBERONC, Instituto de Salud Carlos III, 28029 Madrid, Spain; 3 Department of Radiation Oncology, HUVR, 41013 Seville, Spain; mc.fernandez.fernandez.sspa@juntadeandalucia.es (M.C.F.-F.); mjortizgordillo@yahoo.es (M.J.O.); 4 Department of Medical Oncology, HUVR, 41013 Seville, Spain

**Keywords:** PAI-1/Serpin E-1, rectal cancer, chemoresistance, therapy, Pim kinases

## Abstract

Colorectal cancer (CRC) is the third most common cancer worldwide. The standard treatment in locally advanced rectal cancer is preoperative radiation alone or in combination with chemotherapy, followed by adjuvant chemotherapy. Rectal cancer is highly lethal, with only 20% of patients showing a complete remission (by RECIST) after standard treatment, although they commonly show local or systemic relapse likely due to its late detection and high chemotherapy resistance, among other reasons. Here, we explored the role of PAI1 (Serpin E1) in rectal cancer through the analyses of public patient databases, our own cohort of locally advanced rectal cancer patients and a panel of CRC cell lines. We showed that *PAI1* expression is upregulated in rectal tumors, which is associated with decreased overall survival and increased metastasis and invasion in advanced rectal tumors. Accordingly, *PAI1* expression is correlated with the expression of (Epithelial-to-Mesenchymal Transition) EMT-associated genes and genes encoding drug targets, including the tyrosine kinases PDGFRb, PDGFRa and FYN, the serine/threonine kinase PIM1 and BRAF. In addition, we demonstrate that cells expressing PAI1 protein are more sensitive to the PIM inhibitor AZD1208, suggesting that PAI1 could be used to predict response to treatment with PIM inhibitors and to complement radiotherapy in rectal tumors.

## 1. Introduction

Colorectal cancer (CRC) is the third most common cancer worldwide (10.2%), and the second in Europe (12%). Specifically, cancers of the rectum and rectosigmoid junction represent the 30% of all diagnosed CRC cases. In 2018, there were approximately 200,000 new cases of diagnosed rectal cancer in Europe [1]. Rectal cancers comprise tumors arising within 15 cm of the anal verge. Although these tumors are histologically similar to global CRC tumors, their anatomical situation, invasive growth pattern, surgical approach and treatment outcomes make them a different entity [2]. The standard treatment for patients with locally advanced rectal cancer is preoperative radiotherapy alone or in combination with chemotherapy. The common chemotherapy agents include 5-Fluorouracil (5-FU) and Oxaliplatin, whose function limits the tumor growth [3,4]. Compared to radiotherapy alone, chemoradiation has shown a greater rate of pathological response with no overall survival advantage [5,6,7,8]. Only 20% of patients with rectal cancer show a pathologic complete response in surgery after preoperative chemotherapy and even responding patients inevitably develop refractory disease [9,10]. Five-year survival in CRC is only 11% in the advanced or metastatic stages [1]. Development of chemotherapy resistance is responsible for most of the relapses after surgery and, therefore, patient overall survival. Therefore, it is imperative to explore new targets or biomarkers to stratify patients with advanced or metastatic rectal cancer and predict their response in order to overcome chemotherapy resistance.

Plasminogen Activator Inhibitor 1 (PAI1, also known as SERPINE1), belongs to the serpin super-family and is an inhibitor of tissue plasminogen activator (tPA) and urokinase plasminogen activator (uPA). Therefore, PAI1 is essential for fibrinolysis control and its high levels have been related to the formation of blood thrombotic events due to its anti-fibronolytic activity [11]. Moreover, deregulation of *PAI1* expression has been involved in cardiovascular diseases, obesity, metabolic syndrome and various types of cancer [12]. *PAI1* expression levels depend on the type of cancer. Although bladder urothelial carcinoma and testicular germ cell tumors do not show differences in *PAI1* expression between normal and tumoral tissues, it is significantly increased in other cancers including stomach adenocarcinoma, head and neck squamous cell carcinoma, esophageal carcinoma or thymoma (The Cancer Genome Atlas (TCGA); [13]). Patients with acute leukemia, breast cancer or hepatocarcinoma show an increase in the plasma levels of PAI1 [14,15,16], which are also associated with histological grade of endometrial cancer. [17]. Moreover, PAI1 expression is also correlated with poor outcome in several other cancer subtypes, such as node-negative breast cancer and ovarian serous carcinoma [18,19]. The effect of PAI1 in invasion and metastasis is not clearly defined; while *PAI1* overexpression was significantly associated to those events in osteosarcoma, lung, breast and head-and-neck cancer [20,21,22], it inhibits cell migration and invasion in pancreatic cancer, glioma and melanoma [23,24]. In contrast to the pro-angiogenic role of PAI1 in physiological conditions, its overexpression in tumoral tissues has an anti-angiogenic function [25]. The effect on proliferation is variable, since PAI1 inhibits proliferation in prostate cancer [26] but increases the tumor size of Hela xenografts, fibromatosis or pheochromocytoma [27,28,29]. Besides, several studies have reported the role of PAI1 as anti-apoptotic in Head-and-Neck Cancer Cells (HNCC), ovarian or breast cancer [19,30]. Finally, it has been recently described that PAI1 could have a role promoting inflammation in Non-Small-Cell Lung Carcinoma (NSCLC) [31]. Therefore, it is generally accepted that PAI1 has a role in cancer development, especially in breast cancer where it has been validated clinically [32], but the specific functions and roles of PAI1 depend on the type of cancer.

In this work, we explored the potential of PAI1 as a marker in rectal cancer through the analyses of several public patient databases, as well as our own cohort of locally advanced rectal cancer patients after preoperative radiotherapy. Our data showed that *PAI1* expression is upregulated in rectal tumors, which is associated with decreased survival and increased metastasis in advanced rectal tumors. Accordingly, we observed that *PAI1* expression is correlated with the expression of EMT-associated genes and genes encoding drug targets of tyrosine kinases, PIM1 kinase and BRAF. Using a panel of CRC cell lines, we demonstrated that cells expressing PAI1 are sensitive to Pim inhibitor AZD1208, suggesting that *PAI1* expression could be used as a potential marker effectiveness to treatment with Pim inhibitors after radiotherapy.

## 2. Materials and Methods

### 2.1. Ethics Approval and Consent to Participate

All methods were performed in accordance with the relevant guidelines and regulations of the Institute for Biomedical Research of Seville (IBIS) and University Hospital Virgen del Rocio (HUVR). The entire procedure of patient cohort were performed according to the experimental protocol approved by HUVR Animals Ethics (CEI 0309-N-15). All patients involved in our study provided written informed consent for publication. All tissue samples and patients information were treated according to the Declaration of Helsinki.

### 2.2. Patient Cohort

The entire procedure was approved by the local ethical committee of the HUVR (CEEA O309-N-15). The patient cohort used in this study was previously described [33]. Briefly, tissue samples from 135 patients with locally advanced rectal cancer who received preoperative chemoradiotherapy in the same institution from 2005 to 2014 (Table S1) was obtained from the biobank of HUVR-IBIS (Sevilla, Spain). Eligible patients were those with locally advanced rectal cancer T3-4 N+ M0 (stages II–III) that had completed the neoadjuvant chemoradiotherapy plan before resection with curative intention. Patients with synchronous metastases at diagnosis or with metastatic disease before treatment were excluded. All patients received the same neoadjuvant chemoradiotherapy [33].

### 2.3. Immunohistochemistry Assays

Tumor samples from HUVR were obtained from surgical resection of rectal cancer performed, stored in TMA blocks. Briefly, four-micrometer-thick tissue sections from paraffin blocks were dewaxed in xylene and rehydrated in a series of graded alcohols. Sections were immersed in 3% H_2_O_2_ aqueous solution for 15 min. to exhaust endogenous peroxidase activity and then covered with 1%-blocking reagent (Roche) in PBS to block nonspecific binding sites. Antigen retrieval was performed with a PT Link instrument (Agilent), using citrate/EDTA buffer. Sections were incubated with primary antibody overnight at 4 °C in a humid chamber. Peroxidase-labeled secondary antibodies (visualization reagent-HRP, Dako) and 3,3-diaminobenzidine were applied to develop immunoreactivity, according to the manufacturer’s protocol. Slides were then counterstained with hematoxylin and mounted in DPX (BDH Laboratories, Poole, UK). Sections in which primary antibody was omitted were used as negative controls. Immunostaining was evaluated independently by two observers. The percentage of immunostained tumour cells was scored as follows: 0, negative; 1, < 10%; 2, 10–49% and 3, > 50%.

### 2.4. Cell Culture

Cells were obtained from The European Collection of Authenticated Cell Cultures (ECACC) and cultured according to the manufacturer’s instructions. Briefly, SW48, SW480, T84, LS180, LOVO, HT29 HCT116 and COLO205 were cultured in the corresponding medium and incubated at 37 °C in 5% CO_2_ in a humidified atmosphere. The features of the cell lines used in this study are shown in Supplementary Table S2. All cell lines were authenticated and regularly tested for mycoplasma.

### 2.5. Cytotoxic MTT Assay.

A total of 2 × 10^4^ cells were seeded in 96-well plates and treated with Desatinib, Vemurafenib, or AZD1208 after 24 h. 96 h later, cell viability was measured by the MTT assay and confirmed by crystal violet staining using a iMark Microplate reader (BioRad). The IC50 was calculated using GraphPad Prism 7 software (San Diego, CA, USA).

### 2.6. Western Blot Analyses

Western blotting was performed as previously described [33]. Briefly, cells were grown in serum-free medium for 48 h and then both total cell extracts and medium were used to determine the intracellular and extracellular protein levels of PAI1, respectively. For intracellular determination, cells were washed twice with PBS and lysed by sonication in lysis buffer (50 mM Tris-HCl, pH 7.5; 1% NP-40; 1 mM Na_3_VO_4_; 150 mM NaCl; 20 mM Na_4_P_2_O_7_; 100 mM NaF; 1% Na- deoxycholate; 0.1% SDS; 1 mM EDTA; complete phosphatase inhibitor cocktail (Sigma, San Luis, MO, USA) and complete protease inhibitor cocktail (Sigma)). For extracellular determination, medium was filtered using a 10-kDa ultrafiltration tube (Millipore, Burlington, MA, USA) and centrifuged at 3500× *g* at 4 °C for 30 min. Samples were then run on 6–15% SDS-PAGE gels, transferred to Nitrocellulose membranes (Protran BA83, Whatman, GE Healthcare, Chicago, IL, USA) and immunostained. Primary antibodies used were anti-PAI-1 (1:1000; ab66705) and MAb anti-α-tubulin (1:5000; Sigma 9026); secondary antibody was horseradish peroxidase-labeled rabbit anti-mouse (1:5000; Amersham, GE Healthcare, Chicago, IL, USA). Protein visualization was performed using the ECL detection system (Amersham, GE Healthcare, Chicago, IL, USA). Relative PAI1 protein quantification was performed relative to α-tubulin or total protein (Ponceau staining) for intracellular or extracellular protein, respectively. 

### 2.7. Quantification and Statistical Analysis. 

All statistical analyses were performed using the SPSS statistical software, Chicago, IL, USA (v19), as previously described [34]. Statistically significant differences between study groups was assessed using parametric Student’s t-test or non-parametric Mann–Whitney’s t-test, as appropriate, for pairwise comparisons, or parametric ANOVA for multiple comparisons, setting a P-value of 0.05 as the cut-off for statistical significance. Experiments were performed a minimum of three times. Survival data from patient databases were analyzed by the Log-rank Mantel–Cox statistical test.

### 2.8. Analyses of Cancer Patient Databases. 

Meta-analyses of the PAI1 expression levels in tumor and no tumor colorectal samples were performed using the PrognoScan public patient datasets [35], as previously described [34]. Patient survival was analyzed using the R2 Genomics analysis and visualization platform [36], as previously described [37]. Expression data were downloaded and plotted using GraphPad Prism 6.01. Databases with available survival data were used to generate the Kaplan–Meier plots showing patient survival with the scan method, which searches for the optimum survival cut-off based on statistical analyses (log-rank Mantel–Cox test).

## 3. Results

### 3.1. PAI1 is Upregulated in Rectal Tumors and Associated with Reduced Overall Survival

**Figure 1:**
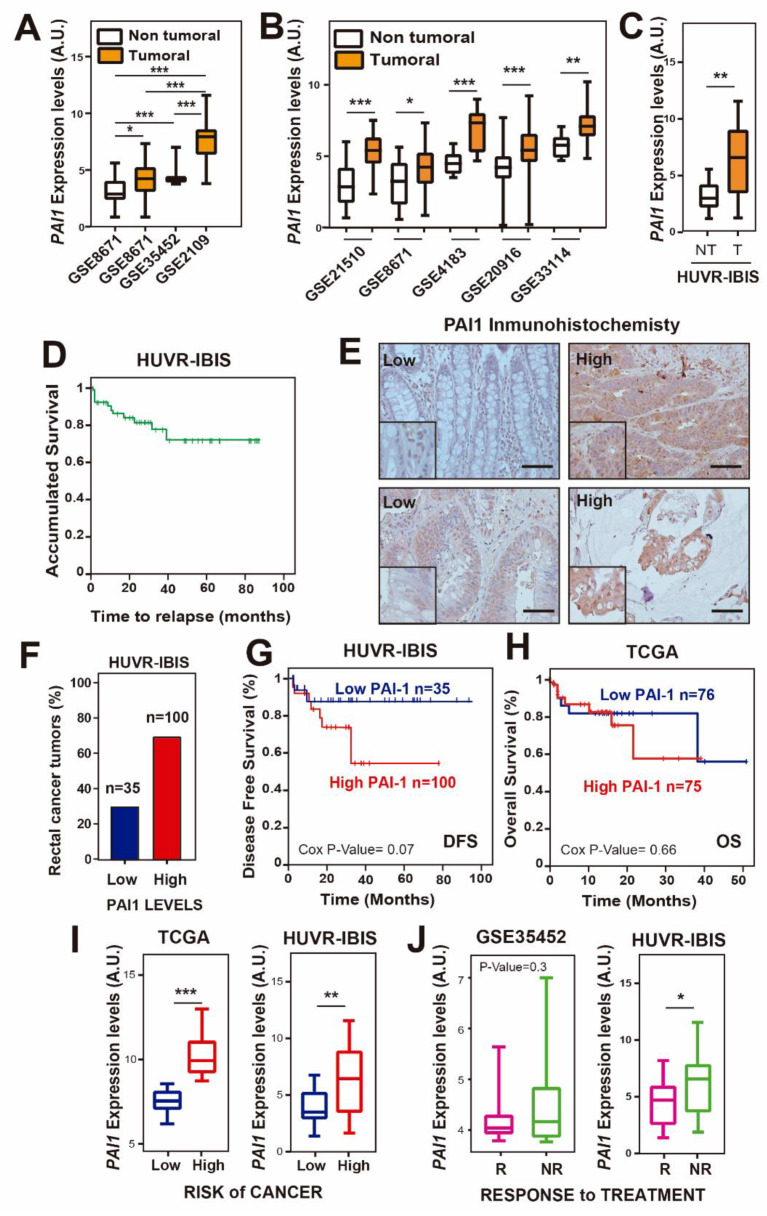
*PAI1* expression is upregulated and reduces overall survival in rectal cancer patients. (**A**) *PAI1* expression in the rectal cancer patient databases GSE35452, GSE8671 and GSE2109. (**B**) *PAI1* expression in colorectal cancer patients from databases GSE21510, GSE8671, GSE4183, GSE20916 and GSE33114. (**C**) *PAI1* expression in rectal patients from the HUVR-IBIS database. For (**A**–**C**), box plots show *PAI1* expression levels in rectal tumor tissue (orange) or non-tumor tissue (white) patients. In (**A**), data were compared using the ANOVA test, except for the GSE35452, whose data were not normally distributed and thus a non-parametric Mann–Whitney’s *t*-test was used. In (**B**,**C**), tumoral and non-tumoral data were compared using Student’s *t*-tests. *p < 0.05; **p < 0.01; ***p < 0.001. (**D**) Overall survival analysis of the HUVR-IBIS rectal tumor cohort (Table S1). (**E**) Representative images of PAI1 immunostaining in rectal cancer tissues. Scale bars, 50 µm. The zoomed region has a magnification factor of 2. (**F**) Percentage of tumors with low or high *PAI1* expression in our HUVR-IBIS cohort of rectal tumor samples (*n* = 135). (**G**) Kaplan–Meier plot showing disease-free survival of patients with high (red) or low (blue) *PAI1* expression levels in our own cohort from HUVR-IBIS. Data were analyzed with the log-rank test, and the associated *P*-values are shown in the graph. (**H**) Kaplan–Meier plot showing overall survival of patients with high (red) or low (blue) *PAI1* expression levels in the TCGA rectal cancer patient database. *P*-values associated with the log-rank test are shown. (**I**) *PAI1* expression levels by risk group in the TCGA rectal cancer patient database (left) and in the HUVR-IBIS cohort (right). (**J**) *PAI1* expression levels in responder (R, pink) and non-responder (NR, green) to treatment patients in the GSE35452 database (left) and in the HUVR-IBIS cohort (right). For (**I**,**J**), data were compared using Student’s *t*-tests. *p < 0.05; **p < 0.01; ***p < 0.001. For (**A**–**C**,**I**,**J**), box plots represent: center line, median; box limits, 25th and 75th percentiles; whiskers, minimum and maximum values.

To study the potential of PAI1 as a marker in rectal cancer, we first analyzed *PAI1* expression levels in four public rectal cancer databases: GSE35452, GSE8671 and GSE2109 (Table S3) [38,39]. Since GSE8671 was the only database containing values of non-tumor tissue and the three databases used the same platform and normalization methods, we used them as a control. We found that the *PAI1* mRNA levels were significantly higher in tumor samples than in normal rectal tissue (Figure 1A). We extended this analysis to colorectal cancer patients in GSE8671 and four additional databases with paired non-tumor and tumor samples (GSE21510, GSE4183, GSE201916 and GSE33114). For all of them, we observed a statistically significant increase of *PAI1* expression in tumor samples (Figure 1B; Table S3). Moreover, this result was corroborated using 135 samples from our previously published cohort of locally advanced rectal cancer patients (HUVR-IBIS) (Figure 1C), who had received preoperative chemoradiotherapy and showed a 5-year overall survival (OS) of 75% (Figure 1D; Table S1) [33], similar to other published datasets [38,39,40]. In this cohort, we also analyzed the levels of PAI1 protein by immunohistochemistry (Figure 1E), defining a high PAI1 protein level in the tumor samples when the score of the stained tumor was higher than 1 on a scale of 0–3 (see Material and Methods) compared with non-tumor samples, and low PAI1 protein level when the score was lower than or equal to 1 [41,42]. Under these conditions, 35 (29.6%) tumor samples showed low expression, and 100 (70.4%) tumor samples showed high expression of *PAI1* (Figure 1F). 

Then, we tested the relevance of *PAI1* expression levels in the survival of patients. We found that patients in our cohort with higher *PAI1* expression showed a decrease in disease-free survival (DFS) compared to that in patients with lower *PAI1* expression, although it did not reach statistical significance (Figure 1G; *p* = 0.076). This was not observed when we analyzed the overall survival data in rectal adenocarcinoma samples from the TCGA database (Figure 1H). However, in patients from this database we observed that individuals with higher levels of *PAI1* were at a higher risk of colorectal cancer than those with lower levels of *PAI1* (*p* < 0.001), a result that was also observed in the HUVR-IBIS database (*p* < 0.01) (Figure 1I). Accordingly, the analysis of the patient response in the HUVR-IBIS cohort showed that non-responder patients had higher levels of *PAI1* (*p* < 0.05), while a non-statistically significant trend was observed in database GSE35452 (*p* = 0.3) (Figure 1J). Altogether, these results suggest that *PAI1* is upregulated in rectal tumors and may be associated with reduced DFS, and that *PAI1* could determine the response to treatment in rectal cancer, at least for the HUVR cohort.

### 3.2. PAI1 Expression Correlates with EMT-Associated Genes in Rectal Tumors

**Figure 2:**
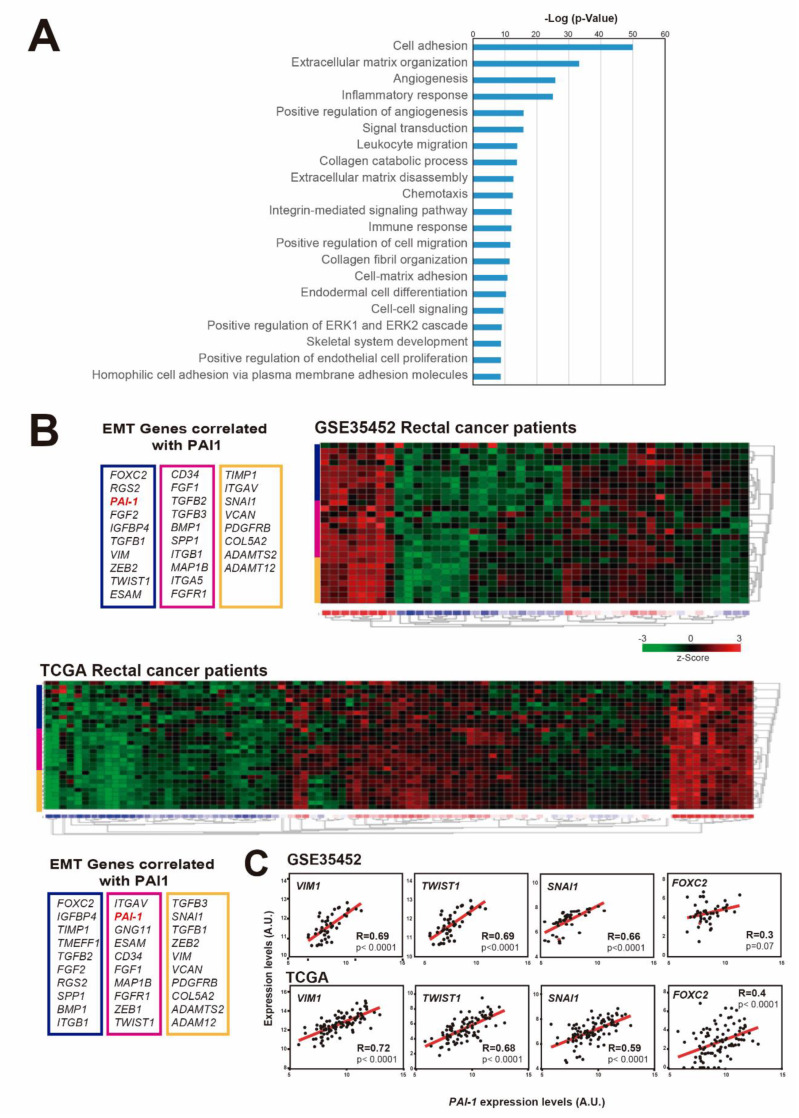
*PAI1* expression correlates with EMT-associated genes in rectal cancer patients. (**A**) Gene Ontology term enrichment analyses of the genes whose expression levels were correlated with those of *PAI1* in the TCGA rectal cancer patient database. (**B**) Heatmaps showing the expression z-scores of EMT-associated genes that correlated with *PAI1* in GSE35452 and TCGA rectal cancer patient databases. (**C**) Correlation of the expression levels of the EMT- associated genes *VIM1, TWIST, SNAI-1* and *FOXC2* with the expression levels of *PAI1* in the GSE35452 and TCGA rectal cancer patient databases (46 and 94 observations, respectively). Data were analyzed using Pearson’s R correlation.

According to previous results in other types of cancer, including breast, head and neck carcinoma and esophageal carcinoma, PAI1 is involved in invasion and metastasis, angiogenesis and proliferation, and it confers a bad prognosis in cancer [43]. To study its role in rectal cancer, we first searched for genes whose expression was correlated with PAI1 expression in rectal tumor samples from the TCGA database. We found that the expression of 2024 genes correlated with that of PAI1 (R < −0.35 or R > 0.35). Gene Ontology term enrichment analyses of these genes showed biological processes associated with cell adhesion, angiogenesis, inflammatory/immune response, cell migration and differentiation (Figure 2A and Supplementary Dataset), which was in agreement with the well-known function of PAI1 as a regulator of these processes.

The epithelial-to-mesenchymal transition (EMT) is a process by which epithelial cells lose their polarity and cell-cell adhesion, and increase migratory and invasive properties to become mesenchymal stem cells. Therefore, we wondered whether *PAI1* expression could correlate with the expression levels of EMT-associated genes in rectal tumors. To explore this possibility, we visualized in a heatmap the expression levels of EMT-associated genes that significantly correlated with *PAI1* expression in two representative patient databases with available microarray expression data: GSE35452 and TCGA (Figure 2B) (Note that microarray data are not available for the HUVR-IBIS cohort). We observed a clear correlation between *PAI1* expression and EMT-associated genes in both rectal tumor databases (Figure 2B), suggesting a relation between *PAI1* expression and the EMT.

Accordingly, the expression levels of *VIM1, TWIST1, FOXC2* and *SNAI1*, the most representative EMT-associated genes, were significantly correlated with *PAI1* expression (Figure 2C). Therefore, these data suggest that the upregulation of *PAI1* in rectal tumors is correlated with the upregulation of EMT-associated genes, which could explain the higher metastasis and tumorigenesis of tumors expressing high levels of *PAI1*.

### 3.3. Upregulation of PAI1 is Associated with Metastasis and Invasion in Rectal Tumors

**Figure 3:**
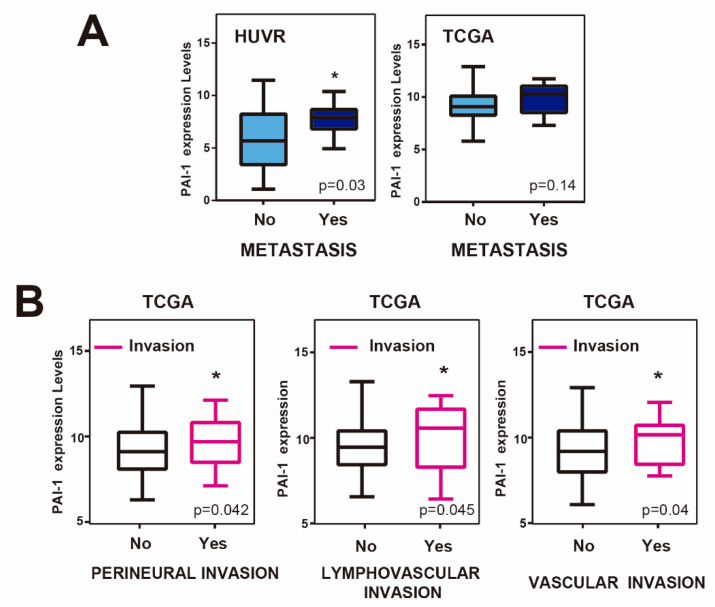
PAI-1 expression, invasion and metastasis in rectal cancer patients. (**A**) *PAI1* expression levels in samples of patients with (YES) or without (NO) metastasis in our own cohort from HUVR-IBIS and the TCGA rectal cancer patient database. (**B**) *PAI1* expression levels in samples of patients with (YES) or without (NO) perineural, lymphovascular or vascular invasion from the TCGA rectal cancer patient database. Student’s *t*-test. *p < 0.05.

Next, we wondered whether *PAI1* expression levels were associated with metastasis in rectal tumors. For this, we first analyzed *PAI1* expression levels in rectal adenocarcinoma patients from our cohort with and without metastasis, finding that it was significantly higher in patients with metastasis (Figure 3A). This tendency was not statistically significant for the TCGA database. However, rectal tumor samples from TCGA showing perineural, lymphovascular or vascular invasion showed significantly higher levels of PAI1 expression (Figure 3B), suggesting that PAI1 may play a role in metastasis and invasion in rectal cancer.

Invasion and metastasis-dependence on PAI1 may be responsible for the bad prognosis of the rectal tumors. Therefore, it will be interesting to suggest possible therapeutic targets to eliminate these malignant phenotypes.

### 3.4. PAI1 Expression Correlates with Drug Target Genes in Rectal Tumors

**Figure 4:**
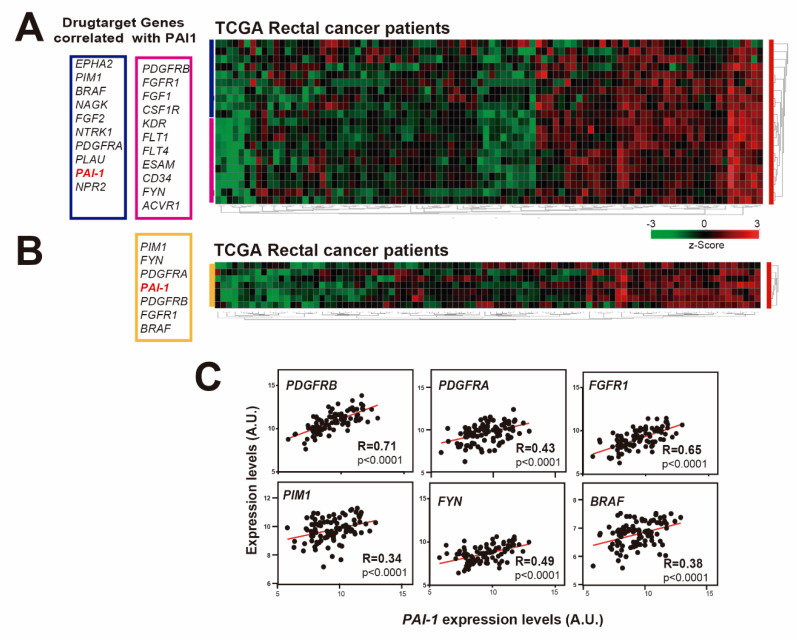
*PAI1* expression correlates with drug target genes in rectal cancer patients. (**A**) Heatmap showing the expression z-scores of drug target genes that correlate with *PAI1* in the TCGA rectal cancer patient database. (**B**) Heatmap showing the z-scores of the most significant drug target genes that correlated with *PAI1* in the TCGA rectal cancer patient database. (**C**) Correlation of the expression levels of the drug targets genes *PDGFRa, PDGFRb, FGFR1, PIM1, FYN* and *BRAF* with the expression levels of *PAI1* in the TCGA rectal cancer patient databases. Data were analyzed using Pearson´s R correlation.

We have shown so far that patients of the HUVR-IBIS cohort with tumors showing upregulated *PAI1* expression have a worse prognosis and more resistance to treatment in rectal cancer (Figure 1G,I–J). An important part of chemotherapy is based on the use of drugs or molecules that inhibit or activate specific targets in tumor cells. Most drug targets are members of phylogenetically conserved protein families and includes G protein-coupled receptors, protein kinases, nuclear hormone receptors, serine proteases and ion channels. Therefore, we analyzed drug target genes whose expression correlated with *PAI1* expression in rectal adenocarcinoma patients from the TCGA database, since microarray data were not available for the HUVR-IBIS cohort (Figure 4A). Among the found drug target genes, we observed highly significant positive correlations for some of the most representative drug target genes used in the treatment of rectal cancer (Figure 4B).

These included platelet-derived growth factor receptors (PDGFRs): *PDGFRB* (R = 0.71, *p* < 0.0001; Figure 4C) and *PDGFRA* (R = 0.43, *p* < 0.0001; Figure 4C). PDGFRs are cell surface type III receptor tyrosine kinases that have been shown to increase proliferation and migration in several tumors [44,45,46,47,48]. *PDGFR*-α and *PDGFR*-β are expressed in CRC tissues and these factors were revealed to stimulate invasion and liver-metastasis formation in mice, having been related with recurrence in this type of cancer [49,50]. *FGFR1* (R = 0.61, *p* < 0.0001; Figure 4C) is a member of the Fibroblast Growth Factor family whose alterations have been recently identified as likely mechanisms of primary and secondary resistance to therapy using anti-EGFR antibody in CRC [51].

The proto-oncogene serine/threonine-protein kinase *PIM1* (R = 0.34, *p* < 0.0001; Figure 4C) is overexpressed in many types of cancers, including CRC, leading to tumor development and progression [52,53,54,55]. The *FYN* gene (R = 0.49, *p* < 0.0001; Figure 4C) encodes a membrane-associated tyrosine kinase that has been involved in the control of cell growth and in the regulation of EMT and metastasis in CRC [56]. Finally, the *BRAF* gene (R = 0.38, *p* < 0.0001; Figure 4C) encodes a serine/threonine kinase and BRAF mutations have been associated with poor prognosis and less response to treatment in metastatic CRC [57]. Altogether, these results suggest that the worse prognosis and increased resistance to treatment in rectal cancer patients with upregulated *PAI1* may be linked to the overexpression of several drug targetable genes.

### 3.5. Resistance to AZD1208 is Associated with PAI1 Expression Levels in Rectal Tumors.

To explore this point, we searched for inhibitors specific for the main lines of targetable proteins correlating with *PAI1* expression. We selected Dasatinib, vemurafenib and AZD1208, inhibitors of broad-spectrum tyrosine kinases, BRAF and Pim Ser/Thr kinases, respectively. Dasatinib is a multitargeted kinase inhibitor proven to be effective for the treatment of several malignancies, including several types of cancers such as CRC [58]. Dasatinib inhibits a broad spectrum of kinases, including Kit, PDGFR, FGFR1 and many others, although it is relatively specific for the Src and Abl family kinases such as Fyn, Yes, Src, and Lyk [59]. As shown by several preclinical studies, Src inhibitors like dasatinib are able to overcome chemoresistance and resistance to targeted agents, such as the EGFR monoclonal antibody cetuximab [60,61,62,63]. Vemurafenib is the first BRAF serine-threonine kinase protein inhibitor authorized for the treatment of adult patients with unresectable or metastatic melanoma with a BRAF V600E positive mutation. Mutations in the BRAF gene substitute the amino acid valine for glutamic acid at position 600 and lead to the oncogenic activation of BRAF proteins. AZD1208 is a potent and selective inhibitor that affects all three isoforms of serine-threonine Pim kinases.

**Figure 5:**
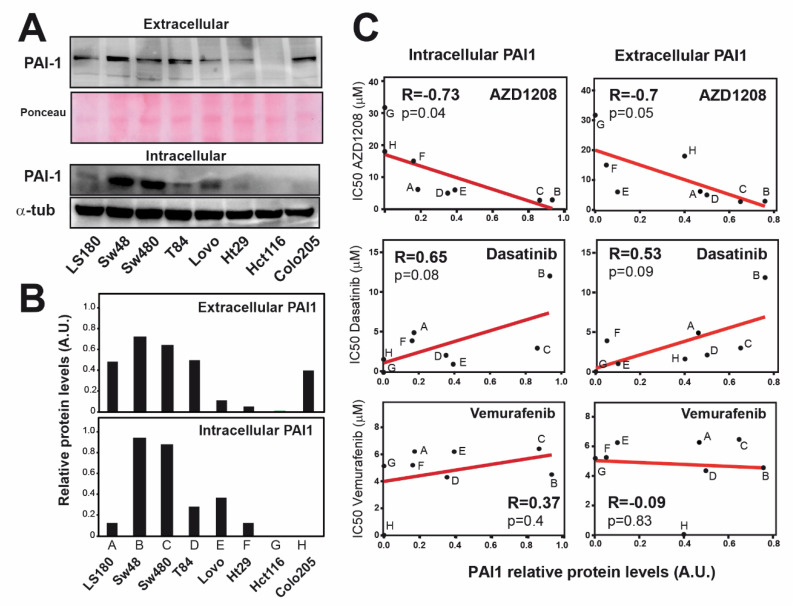
PAI1 expression correlates with sensitivity to the PIM inhibitor AZD1208 but not with Tyr Kinases inhibitor Dasatinib nor B-Raf inhibitor vemurafenib. (**A**) Intracellular and extracellular protein levels of PAI1 in CRC cell lines by Western blot. α-tubulin (α-tub) is used as the intracellular loading control, and total protein as the extracellular control. (**B**) Quantification of PAI1 protein levels from (**A**) relative to α-tub for intracellular quantification or to total protein for extracellular quantification (total protein measured with Ponceau staining). (**C**) Scatter plots showing the IC_50_ value (concentration that induces a 50% of cell death) to Vemurafenib, Dasatinib or the PIM inhibitor AZD1208 for the cell lines in (**A**), versus the PAI1 protein levels from (**B**). The Pearson’s *R* correlation coefficient is shown with the associated *P*-value. IC_50_ values plotted in C are the average of three independent experiments. The range of concentration for the drugs is 0–300 µM.

We wondered whether *PAI1* expression levels could determine the resistance to dasatinib, vemurafenib or AZD1208. To test this, we first analyzed the expression levels of PAI1 in a panel of eight CRC cell lines by Western blot, both intra and extracellular, showing a high variability among them (Figure 5A–B). Next, these cells were treated with different doses of dasatinib, vemurafenib and AZD1208 to calculate the IC_50_ using the MTT assay as a readout of their sensitivity. We detected similar sensitivity/resistance behavior to the B-Raf inhibitor vemurafenib in all cell lines independently of PAI levels (Figure 5C; Table S4). For dasatinib, we found more heterogeneous results, although sensitivity was not correlated with PAI1 protein levels either (Figure 5C; Table S4). Interestingly, the analysis with the Pim inhibitor AZD1208 showed that, although the IC_50_ was negatively correlated with PAI1 protein levels, most of the cell lines expressing PAI1 were similarly affected by AZD1208 treatment, in contrast to cell lines expressing undetectable levels (Figure 5C; Table S4), indicating that PAI1 expression determines sensitivity to AZD1208-mediated PIM inhibition. The mutation status of *KRAS*, *BRAF* and *PI3KCA* did not seem to be related to the degree of sensitivity of these cell lines to AZD1208. Therefore, our data may suggest that PIM inhibition may be a suitable treatment for the patients with a worse prognosis due to high levels of PAI1 in rectal tumors.

## 4. Discussion

In Europe, 200,000 new cases of rectal cancer were diagnosed in 2018. Rectal cancer is a highly lethal cancer, with only 20% of patients showing a complete response after surgery and even responding patients inevitably developing refractory disease [1]. This is due to its late detection and to the development of chemotherapy resistance to common treatments. Thus, it is essential to find new biomarkers to stratify patients with advanced or metastatic rectal cancer and predict their response in order to overcome chemotherapy resistance. Here, we found that *PAI1* gene expression is upregulated in rectal cancer and that this upregulation may reduce the DFS and the response to treatment of patients, at least those of our cohort (HUVR-IBIS), and could be used as a bad prognosis marker (Figure 1). Moreover, high expression levels of *PAI1* are associated with increased metastasis and invasion of rectal tumors in this cohort and TCGA database, respectively (Figure 3), which is correlated with EMT-associated and drug target genes expression, including *PDGFRa, PDGFRb, FYN, PIM1* and *BRAF* (Figure 2 and Figure 4). Strikingly, cells expressing PAI1 protein are more sensitive to PIM inhibitor AZD1208 (Figure 5), suggesting that PAI1 could be used to predict increased efficacy of this PIM inhibitor and may complement radiotherapy in rectal tumors.

PAI1 belongs to the family of serine protease inhibitors, also known as serpins, and is the main regulator of the plasminogen activation system, acting by inhibition of tPA and uPA. PAI1 deregulation has been associated with cardiovascular diseases, obesity, metabolic syndrome and various types of cancer [12]. We found that *PAI1* gene expression is upregulated in several CRC public datasets and validated this with our own cohort of patients with advanced rectal cancer (HUVR-IBIS). Patients with higher *PAI1* expression show a decrease in the survival probability in our patient database, as compared to patients with lower *PAI1* expression. This suggests that *PAI1* expression could be a potential independent biomarker for survival. Accordingly, we found that patients with higher *PAI1* expression showed a tendency to respond worse to treatment in the HUVR-IBIS cohort. Moreover, *PAI1* expression is also correlated with poor outcome in several other cancer subtypes, particularly in ovarian serous carcinoma and node-negative breast cancer [18,19]. In fact, PAI1 has been validated clinically in breast cancer as a biomarker [32]. Furthermore, *PAI1* expression is significantly enhanced in some other cancers, including stomach adenocarcinoma, head and neck squamous cell carcinoma, esophageal carcinoma or thymoma [13]. Additionally, an increase in the plasma levels of PAI1 has been shown in acute leukemia, breast cancer, hepatocarcinoma and colon cancer [14,15,16]. However, bladder urothelial carcinoma and testicular germ cell tumors do not show differences in PAI1 expression between normal and tumoral tissue [13], indicating that PAI1 is a common but not universal biomarker of cancer progression. 

We have observed that high expression levels of *PAI1* are associated with increased metastasis and invasion in rectal tumors of the HUVR-IBIS and TCGA cohorts, respectively, and that, accordingly, it is highly significantly correlated with EMT-associated gene expression in the latter database and in the GSE35452 (Figure 2 and Figure 3). In agreement with our results, overexpression of PAI1 was significantly associated with metastasis and invasion in lung, head and neck and breast cancers, as well as osteosarcoma [20,21,22]. In contrast, PAI1 has been shown to have an inhibitory effect over cell migration and invasion in other types of cancer, as it is the case in pancreatic cancer, glioma or melanoma [23,24]. Therefore, it seems that the outcomes of *PAI1* overexpression depends on the cellular context. In line with the pro-metastatic role of PAI1, it has been shown to facilitate invasion and lung metastasis in osteosarcoma cells by promoting MMP13 expression and secretion [20]. Moreover, it has been described that PAI1 and CCL5 signaling in endothelial cells leads to increased metastasis in EMT-induced triple-negative breast cancer cells [22]. Finally, clinical data showed a significant increase in PAI1 levels in plasma of CRC patients with liver metastasis and infiltration [14].

Our analyses using rectal cancer patient public databases showed that non-responder rectal cancer patients had higher expression levels of *PAI1* in the HUVR-IBIS cohort (Figure 1H; GSE35452; [39]). Moreover, we showed that *PAI1* expression positively correlates with the expression of several drug targets genes, including PDGFRa, PDGFRb, FYN, PIM1 and BRAF in the TCGA database (Figure 4). A high number of preclinical studies have demonstrated the ability of Src inhibitors like dasatinib to inhibit most of these drug target genes, as PDGFRa, PDGFRb or FYN, and to overcome chemoresistance in CRC [60,61,62,63,64,65,66,67]. However, clinical trials with dasatinib combined either with conventional chemotherapy or with suppression of EGFR by cetuximab and with FOLFOX, failed to show any meaningful clinical response [68,69]. A recent study showed that this failure in clinical trials is possibly due to the fact that Desatinib reduces apoptosis triggered by 5-FU in colon carcinoma [70]. However, data from our own laboratory suggest that dasatinib may be active in combination with oxaliplatin (but not with 5-FU) only in patients whose tumors show high p-Src levels [66].

Our data suggest that treatment with PIM inhibitors may be beneficial in combination with radiotherapy in patients with tumors showing PAI1 expression. Therefore, PAI1 can be used to select patients for whom this combination therapy could be beneficial. Overexpression of PIM kinases is common in many types of tumors, including CRCs [71,72]. Indeed, both in vitro and in vivo studies have shown that individual PIM kinases are weak oncogenes that can become stronger ones in cooperation with c-MYC in some tumors. These precedents suggest that PIM kinases may be good targets for drug development [73,74]. The pan-PIM kinase inhibitor AZD1208 acts over the serine/threonine kinases PIM1, 2 and 3, which may result in cell cycle arrest and apoptosis when those kinases are overexpressed. We used it as a tool compound to prove that one of the drug target paths correlating with PAI1 expression may be suitable to compensate tumor survival after radiotherapy and poor prognosis. We demonstrated that PAI1 expression leads to sensitivity to AZD1208 PIM inhibitor, suggesting that PAI1 expression could be used as a prognosis marker to stratify patients with bad prognosis of the rectal tumors and predict increased efficacy of this PIM inhibitor. 

## 5. Conclusions

Our study shows that *PAI1* expression is upregulated and correlates with decreasing survival and increasing metastasis and invasion. Moreover, we propose PAI1 as a new predictive marker to stratify patients according to their response to standard treatment in rectal cancer. We demonstrate using a panel of CRC cell lines the sensitivity to AZD1208 Pim inhibitors of cells with *PAI1* expression, suggesting that PAI1 could be used as a prognosis marker to stratify rectal cancer patients and predict increased efficacy of this Pim kinase inhibitor in those with a bad prognosis.

## Supplementary Materials

The following are available online at https://www.mdpi.com/2073-4409/9/5/1071/s1. Table S1. Patient cohort characteristics; Table S2. Mutational analysis of the CRC cell lines used in this study; Table S3. Characteristics of patient public databases used in this study. Table S4. Determination of the IC50 value to AZD1208, Dasatinib and Vemurafenib; Supplementary Dataset. Complete Gene Ontology enrichment analysis of genes whose expression is correlated with PAI1.
