# Supplementary material for: PAI1 is a Marker of Bad Prognosis in Rectal Cancer but Predicts a Better Response to Treatment with PIM Inhibitor AZD1208
Source: Cells. 2020 Apr 25;9(5):1071. doi: 10.3390/cells9051071 (PMC7291071; doi:10.3390/cells9051071)
Supplement: Supplementary file 1 [file cells-09-01071-s001.zip › Munoz-Galvan_Supplementary_Material.pdf]

Muñoz-Galván *et al.* "PAI1 is a marker of bad prognosis in rectal cancer but predicts a better response to treatment with PIM inhibitor AZD1208"

Supplementary Material:

Supplementary Table S1

Supplementary Table S2

Supplementary Table S3

Supplementary Table S4

Supplementary Figure S1

Supplementary Table S1: Patient HUVR cohort characteristics.

Cohort description	Cohort<br><br>N=135
**Gender (years)**<br><br>• Women<br>• Men<br>• Age	 <br>43<br>92<br>Median 68 years old
**Tumor localization (distance from the anal verge)**<br><br>• Low rectum (3-5 cm)<br>• Medium rectum (6-9 cm)<br>• High rectum (10-15 cm)<br>• Non recorded in medical history	 <br>54<br>45<br>34<br>2
**Histology and tumoral grade**<br><br>• Adenocarcinoma GI<br>• Adenocarcinoma GII<br>• Adenocarcinoma GIII<br>• Non recorded in medical history	 <br>28<br>67<br>5<br>35
**Pre-treatment stage (TNM)**<br><br>• I<br>• IIA<br>• IIB<br>• IIC<br>• IIIA<br>• IIIB<br>• IIIC<br>• IV	 <br>1<br>23<br>3<br>10<br>7<br>67<br>22<br>2
**Surgery**<br><br>• Low anterior resection<br>• Abdominoperineal resection<br>• Other procedures	 <br>89<br>41<br>5
**Tumor recurrence**<br><br>• No events<br>• Local relapse<br>• Hepatic relapse<br>• Lung relapse<br>• Local and Hepatic relapse<br>• Local and Lung relapse<br>• Local and peritoneal relapse<br>• Distant relapse in other localizations	**Tumor recurrence**<br><br>101<br>11<br>7<br>7<br>2<br>3<br>1<br>3

Supplementary Table S2. Mutational analysis of the CRC cell lines used in this study.

Cell line	Kras mutation	Braf V600E	PI3KCA mutation
COLO205	**Wild type**	**Mutated**	**G914R**
HT29	**Wild type**	**Mutated**	**P449T**
HCT116	**G13D**	**Wild type**	**H1047R**
LS180	**G12D**	**Wild type**	**H1047R**
LOVO	**G13D**	**Wild type**	**ND**
T84	**G13D**	**Wild type**	**E542K; H1047R**
SW480	**G12V**	**Wild type**	**ND**
SW48	**Wild type**	**Wild type**	**G914R**

Supplementary Table S3. Characteristics of patient public databases used in this study.

Databases	N	Sex	Age	histology	Reference
**GSE8671**	NT=32<br>T=32	F=37.5%<br>M=62.5%	38-83<br>20-50=15.6%<br>50-83=84.4%	Non tumoral<br>Adenocarcinoma<br>Rectum	Sabates-Bellver J et al., Mol Cancer Res 2007
**GSE35452**	T=46	nd	0-90	Rectal cancer	
**GSE2109**	T=38	F=18<br>M=20	20-90	Adenocarcinoma<br>Rectum	
**GSE21510**	NT=25<br>T=homogenized 19<br>Lcm=104	F=1/3<br>M=2/3	20-92	CRC	Tsukamoto et al., Clin Cancer Res 2011
**GSE4183**	NT=8<br>T=15	F=94<br>M=51	NT=50.6 +-5.97<br>T=<br>DukeB =65.3 +-13.6<br>Duke C-D=67.5+-11.8	Healthy =8<br>CRC Duke B= 7<br>CRC Duke C-D= 8	Galamba O et al., Dis Markers 2008
**GSE20916**	NT=34<br>T=111	F=94<br>M=51	38-85	Adenocarcinoma de colon	Skrzypczak M et al., PLoS One. 2010
**GSE33114**	NT=6<br>T=90	F=48<br>M=42	34-95	Primary colorectal cancer	de Sousa E Melo et al., Cell Stem Cell. 2011

Supplementary Table S4. Determination of the IC_{50} value (concentration of drug necessary to induce 50% of cell death) to AZD1208, Dasatinib and Vemurafenib. Average and SEM of three independent experiments is shown**.**

**Cell lines**	**IC50 (μM)**		
	**AZD1208**	**Dasatinib**	**Vemurafenib**
**LS180**	7.2 ± 0.7	5.3 ±0.8	6.2 ± 0.8
**Sw48**	2.9 ± 1	12 ± 1.4	4.5 ± 1
**Sw480**	2.8 ± 1.2	3.1 ± 1.2	6.4 ± 0.7
**T84**	6.24 ± 0.7	2.2 ±0.9	4.3 ± 1.3
**Lovo**	6.8 ± 1.2	1.1 ± 0.5	6.2 ± 0.5
**Ht29**	15 ± 0.9	4 ± 1	5.2 ± 0.9
**Hct116**	31.7 ± 2.3	0.11 ± 0.012	5.14 ± 0.8
**Colo205**	18 ± 1.4	1.7 ± 0.2	0.03 ± 0.006

Supplementary Figure S1

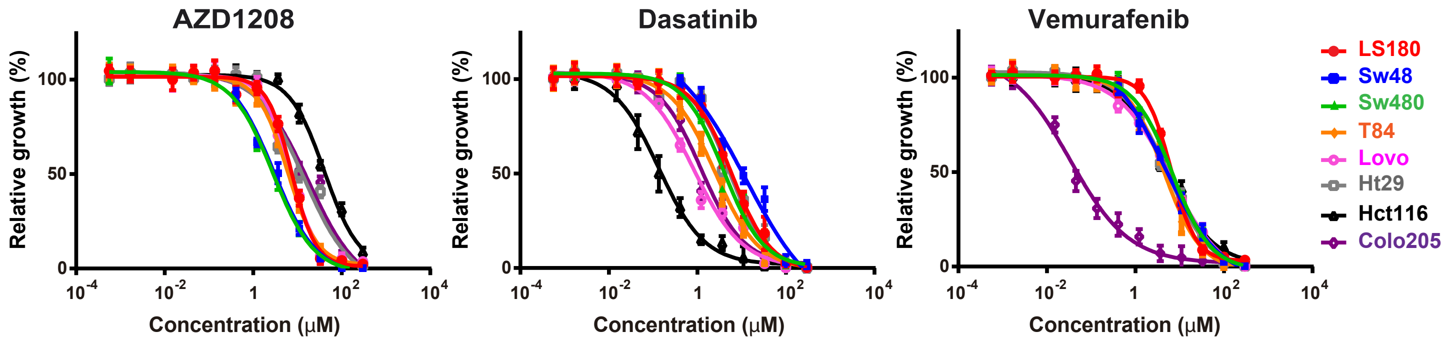

Figure S1. Determination of IC_{50} value. Representative graph of IC_{50} curves (IC_{50} concentration that induces a 50% of cell death) to PIM Inhibitor AZD1208, Dasatinib or Vemurafenib for the cell lines LS180, Sw48, Sw480, T84, Lovo, Ht29, Hct116 and Colo205.
